# Free-breathing radial 3D fat-suppressed T1-weighted gradient echo (r-VIBE) sequence for assessment of pulmonary lesions: a prospective comparison of CT and MRI

**DOI:** 10.1186/s40644-021-00441-3

**Published:** 2021-12-20

**Authors:** Nan Yu, Haifeng Duan, Chuangbo Yang, Yong Yu, Shan Dang

**Affiliations:** 1 Department of Radiology, The affiliated hospital of Shaanxi university of Chinese medicine, Xian Yang, China; 2 Department of Radiology, The affiliated hospital of Shaanxi university of Chinese medicine, -2# Weiyang Western Road, 712000 Xian Yang, China

**Keywords:** computed tomography, lung, Magnetic Resonance Imaging, pulmonary nodules

## Abstract

**Purpose:**

To determine whether the pulmonary MR imaging with free-breathing radial 3D fat-suppressed T1-weighted gradient echo (r-VIBE) sequence can detect lung lesions and display lesion profiles with an accuracy comparable to that of computed tomography (CT), which is the reference standard in this study.

**Population:**

Sixty-three consecutive patients were prospectively enrolled between October, 2016 and March, 2017. All the patients received both 3T MRI scanning with a free-breathing r-VIBE sequence and chest standard CT. Morphologic features of lesions were evaluated by two radiologists with a 5-point system. Chest standard CT were used as reference standard. Weighted kappa analysis and chi-squared test were used to determine both inter-observer agreement and inter-method agreement.

**Results:**

A total of 210 solid pulmonary nodules or masses and 1 ground-glass nodule were detected by CT. Compared to CT, r-VIBE correctly detected 95.7% of pulmonary nodules, including 100% of detection rate with diameter greater than 6 mm, 92.3% of pulmonary nodules with diameter between 4 and 6 mm, and 83.3% of pulmonary nodules with diameter less than 4 mm The inter-method agreements between r-VIBE and standard-dose CT were either “substantial” or “excellent” in the evaluation of following features of pulmonary nodules with diameter more than 10mm: including lobulation, spiculation, convergence of vessels, bubble-like attenuation, cavitation and mediastinal lymph node enlargement (0.605≤K≤1.000; *P*<0.0001). However, K values for inter-method agreements were significant but “moderate” or “poor” for evaluating pleural tag, halo, and calcification (0.355≤ K≤0.451; *P*<0.0001).

**Conclusion:**

The use of pulmonary MR imaging with r-VIBE showed high detection rate of pulmonary nodules and inter-method agreement with CT. It is also useful for nodule morphologic assessment.

## Introduction

Magnetic Resonance Imaging (MRI) of lung is more and more accepted as a valuable additional imaging modality for chest [1–4]. Following several technical advancements in MRI technology, it is now used as a complementary diagnostic tool for pulmonary disease [5, 6]. T2 weighted half-fourier single-shot turbo spin echo (T2-HASTE) sequence can be used to visualize pathological changes [7]; T1 weighted 3D gradient recall echo (T1W-GRE) is best to assess pulmonary nodules and mediastinal disease with breath hold [8]; also ultrashort echo time (UTE) imaging is acceptable for pulmonary nodules detected [9]. For pulmonary solid nodules with a diameter of 5–8 mm, detection rates of pulmonary MRI have been between 60 and 90% in most clinical studies. For lesions with a diameter of 8 mm or more, clinical detection rates have been close to 100% [10–14]. Conventional T1-weighted 3D gradient-echo volumetric interpolated breath-hold examination (C-VIBE) showed highest sensitivity (69%) in detecting lung lesions [15]. However, patients have to hold their breath. which may hardly for those with compromised pulmonary function.

Conventional MRI sequences acquiring k-space data in a line by line (C-VIBE) manner makes them sensitive to motion as the data is acquired contiguously. Respiratory motion may disturbs the phase encoding scheme resulting in artefact. An alternative method of overcoming sensitivity to motion is to change the way k-space is acquired. Radial technique is one of such acquisition schemes, which based on acquiring k-space data along radial spokes. Due to the overlap of spokes in the centre, the distribution of k-space data along individual spokes is averaged out. Because of low sensitivity to motion, radial stack-of-stars acquisition allows a free-breathing T1W sequence that can emulate lesion delineation and filled.

The r-VIBE sequence was reported to have less sensitive to motion compared with the Cartesian acquisition scheme in the conventional VIBE sequence. It can provide high-resolution images, and has been described as a valuable T1-weighted gradient-echo sequence for MRI, which is performed to examine patients who are unable to hold their breath [16–20].

We hypothesized that if pulmonary MR imaging with r-VIBE, it would be possible to detect and visualize the structure of solid pulmonary lesions. Thus, the objective of this study was to determine whether pulmonary MR imaging with r-VIBE can detect pulmonary lesions. In addition, the potential of r-VIBE for morphologic characterization of pulmonary nodules was assessed in comparison with chest CT as the standard of reference.

## Materials and methods

### Subjects

This prospective study was approved by the Institutional Review Board of our hospital. A written informed consent was obtained from all the patients who participated in this study.

Sixty-three consecutive patients were enrolled as their chest CT scans raised suspicions of lung cancer. Pulmonary r-VIBE examinations were also performed on all patients within 48 h after chest CT. Exclusion criteria were as follows: (1) age less than 18 years, (2) pregnancy or breast feeding condition, and (3) contraindication to MRI. (4) Patient with innumerable nodules which cannot be marked. fourteen patients were excluded according to the exclusion criteria mentioned above. Out of the 50 patients included in this study, there were 39 males and 10 females (mean age, 64.1 years old; age range, 48–83 years). Pathological results were obtained by transthoracic needle biopsy or completely surgically resected. Solid pulmonary lesions were finally identified as benign and malignant by referring to following pathological results: 38 lung cancer cases (19 cases of adenocarcinoma, 5 cases of squamous cell carcinoma, 6 cases of small cell carcinoma, and 1 case of giant cell carcinoma; 2 cases of carcinoid tumor, 4 cases of metastases, and 1 case of adenosquamous carcinoma), 3 chronic inflammation cases, 2 organizing pneumonia cases, 1 tuberculoma case, 1 inflammatory pseudotumor case, 1 pulmonary sequestration case, 1 pulmonary abscess case, and 1 pneumonia case. One ground-glass nodule was diagosised as adenocarcinoma by surgically resected sample. Ten patients with lung cancer had more than one nodules, and the remaining nodules were diagnosed as metastases lesions according to imaging features and follow-up results.

### Multi-slice Computed Tomography (CT)

Using a 64-row CT scanner (Bright Speed, respectively; General Electric, Milwaukee, WI, USA). No contrast medium was used to obtain CT scans. Images were obtained using following conditions: 1.25 mm collimation, 27.5 mm table feed per rotation, 0.6 s/rot, 165 mA tube current, and 120 KV tube voltage. Images were reconstructed in axial and coronal orientations with 1mm slice thickness.

### Magnetic resonance imaging

To perform MRI studies, a 3.0-T SKYRA MR scanner (MAGNETOM 3.0T SKYRA MR scanner, Siemens Healthcare, Erlangen, Germany) was operated with explorer gradients (maximum gradient of 40, 40, 45 m T/m along x, y and z axis, respectively, and a slew rate of 200 m T/m/ms) and a phased-array multi-coil system (12 elements). Images were obtained using a free-breathing r-VIBE sequence (TR, 2.79ms; TR/TEs, 1.39ms; Flip (st), 5; FOV, 380mm; Matrix, 320 × 320; Slice thickness, 1.2mm; Breath-hold, no; Acquisition time (min), 5:30). The r-VIBE sequence was acquired with a totally free-breathing method, which did not involve any navigator or trigger.

### Image quality evaluation

An image subjective quality evaluation was performed on pulmonary MR imaging and r-VIBE sequence. For evaluation of image quality, a 5-point visual scoring system was based on the following parameters: vessel edge sharpness, lesion clarity, and respiration artifact (1, worse; 2, poor; 3, acceptable; 4, good; 5, excellent). Two radiologists independently evaluated image quality, but final score was obtained by consensus of the two radiologists. For each method, image quality was evaluated at the level of lung apices, aortic arch, carina, left atrium, and lung bases.

### Reference standard for radiological findings on standard-dose CT

To evaluate radiological findings of pulmonary MR imaging and r-VIBE sequence, standard-dose CT images of each patient were reviewed by two radiologists with 10 and 15 years of experience. For each patient, final evaluation was done by consensus of the two readers. Final results were used as reference standard for radiological findings in this study.

### Lesion detection

After obtaining images with standard-dose CT and r-VIBE (MRI) sequence, we evaluated them on the same PACS (Picture Archiving and Communication System). MRI images were first analyzed followed by MSCT images. For each reader, reading interval between CT and MR images was more than one week. For each review, radiologists were asked to record and mark each visible nodule or mass. For any detected lesion, the location was noted and the longest diameter was measured. The final largest diameter was obtained by averaging the results from the two radiologists.

### Lesion profiles display

For each nodule or mass, the capability of lesion profiles displayed by r-VIBE (MRI) sequence were compared with those of thin-section CTs using a 5-point visual scoring system (1, absent; 2, probably absent; 3, equivocal; 4, probably present; 5, present). Thus, we determined the morphological characteristics of each lesion (Shape, peripheral structure, internal characteristics, visceral pleural and mediastinal lymph node). For each radiological finding, final score was determined by consensus of the two radiologists.

### Statistical analysis

Detection rate of MRI sequence was defined as the ratio of nodules or masses detected by MRI to all the nodules identified in CT scans.

Weighted kappa analysis and x^2^ test were used to determine inter-observer agreement for assessment of subjective image quality and lesion profiles’ display, which were obtained by standard-dose CT as well as pulmonary MR imaging with r-VIBE sequence. Inter-observer agreement was graded as follows: poor for *K* < 0.21, fair for *K =* 0.21–0.40, moderate for *K =* 0.41–0.60, substantial for *K =* 0.61–0.80, and excellent for *K =* 0.81–1.00.

## Results

### Image quality evaluation

Table 1 displays inter-observer agreement on quality evaluation of images obtained with r-VIBE between two readers. All inter-observer agreements were significant and considered as “substantial.” ( K = 0.887; *P* < 0.0001).

**Table 1:** Interobserver agreement for assessment of image quality between two readers

		Visual score						
**Methods**	**observers**	**1**	**2**	**3**	**4**	**5**	**Kappa value**	***P*value**
**MR imaging with r-VIBE**	Reader 1	0	0	8	16	23	0.887	<0.0001
	Reader 2	0	0	6	18	23		

### Lesion detection

A total of 210 solid pulmonary nodules or masses and 1 ground-glass nodule were detected by CT. The lesion sizes were classified according to Fleischner Society guidelines. There were 47 lesions whose maximum diameter was greater than 10 mm (22.3%); the diameter of 82 (38.9%) lesions was between 6 and 10 mm; the diameter of 52 (24.6%) lesions was between 4 and 6 mm; and the diameter of 30 (14.2%) lesions was less than 4 mm. Compared to CT, r-VIBE correctly detected 95.7% of pulmonary nodules. As shown in Table 2, r-VIBE (MRI) had 100% detection rate for pulmonary nodules with a maximum diameter of more than 6 mm, and 92.3% of pulmonary nodules with diameter between 4 and 6 mm. Moreover, r-VIBE detected 83.3% of pulmonary nodules, which had a diameter of less than 4 mm. Four nodules detected on r-VIBE were not detected by CT, all of which were in the section of pulmonary vessels.

**Table 2:** Detection ability of r-VIBE (MRI) for pulmonary nodules

	Number of nodules (n)		Detection rate
Nodule Size((mm)*	**CT(+)/MRI(+)**	**CT(+)/MRI(-)**	
**>10mm**	**47**	**0**	**100%**
**>6-10 mm**	**82**	**0**	**100%**
**>4-6 mm**	**48**	**4**	**92.3%**
**≤4 mm**	**25**	**5**	**83.3%**

* Average of length and width

### Morphological characteristics

To further analyze morphological characteristics, we used 47 lesions whose maximum diameter were greater than 10mm. Table 3 showed all inter-observer agreements for the assessment of lung lesion profiles. All inter-observer agreements were significant and considered as “substantial” or “excellent.”

**Table 3:** Inter-observer agreement for assessment of morphological characteristics obtained with the two methods

			Visual score						
**morphological characteristics**	**Methods**	**observers**	**1**	**2**	**3**	**4**	**5**	**Kappa value**	***P*value**
**Lobulation**	Standard-dose CT	Reader 1	26	0	0	0	21	0.957	<0.0001
		Reader 2	25	0	0	0	22		
	MR imaging with r-VIBE	Reader 1	27	0	2	0	18	0.871	<0.0001
		Reader 2	25	0	0	0	22		
、**Spiculation**	Standard-dose CT	Reader 1	16	0	0	0	31	0.953	<0.0001
		Reader 2	17	0	0	0	30		
	MR imaging with r-VIBE	Reader 1	20	0	4	11	12	0.873	<0.0001
		Reader 2	18	1	5	11	12		
**Pleural indentation**	Standard-dose CT	Reader 1	23	0	0	0	24	0.957	<0.0001
		Reader 2	22	0	0	0	25		
	MR imaging with r-VIBE	Reader 1	28	0	3	10	6	0.948	<0.0001
		Reader 2	28	0	3	10	6		
**Bubble-like attenuation**	Standard-dose CT	Reader 1	36	0	0	0	11	1.00	<0.0001
		Reader 2	36	0	0	0	11		
	MR imaging with r-VIBE	Reader 1	37	0	2	2	6	0.924	<0.0001
		Reader 2	37	1	3	1	5		
**Convergence of vessels**	Standard-dose CT	Reader 1	25	0	0	0	22	0.915	<0.0001
		Reader 2	23	0	0	0	24		
	MR imaging with r-VIBE	Reader 1	30	0	0	3	14	0.941	<0.0001
		Reader 2	30	0	0	2	15		
**Halo**	Standard-dose CT	Reader 1	43	0	0	0	4	0.846	<0.0001
		Reader 2	44	0	0	0	3		
	MR imaging with r-VIBE	Reader 1	45	1	0	0	1	1.00	<0.0001
		Reader 2	45	1	0	0	1		
**Cavitation**	Standard-dose CT	Reader 1	42	0	0	0	5	1.00	<0.0001
		Reader 2	42	0	0	0	5		
	MR imaging with r-VIBE	Reader 1	42	0	0	0	5	1.00	<0.0001
		Reader 2	42	0	0	0	5		
**Calcification**	Standard-dose CT	Reader 1	43	0	0	0	4	0.877	<0.0001
		Reader 2	42	0	0	0	5		
	MR imaging with r-VIBE	Reader 1	45	0	0	0	2	1.00	<0.0001
		Reader 2	45	0	0	0	2		
**Mediastinal lymph node enlargement**	Standard-dose CT	Reader 1	29	0	0	0	18	1.00	<0.0001
		Reader 2	29	0	0	0	18		
	MR imaging with r-VIBE	Reader 1	24	0	1	3	19	0.989	<0.0001
		Reader 2	24	0	1	2	20		

**Fig. 1:**
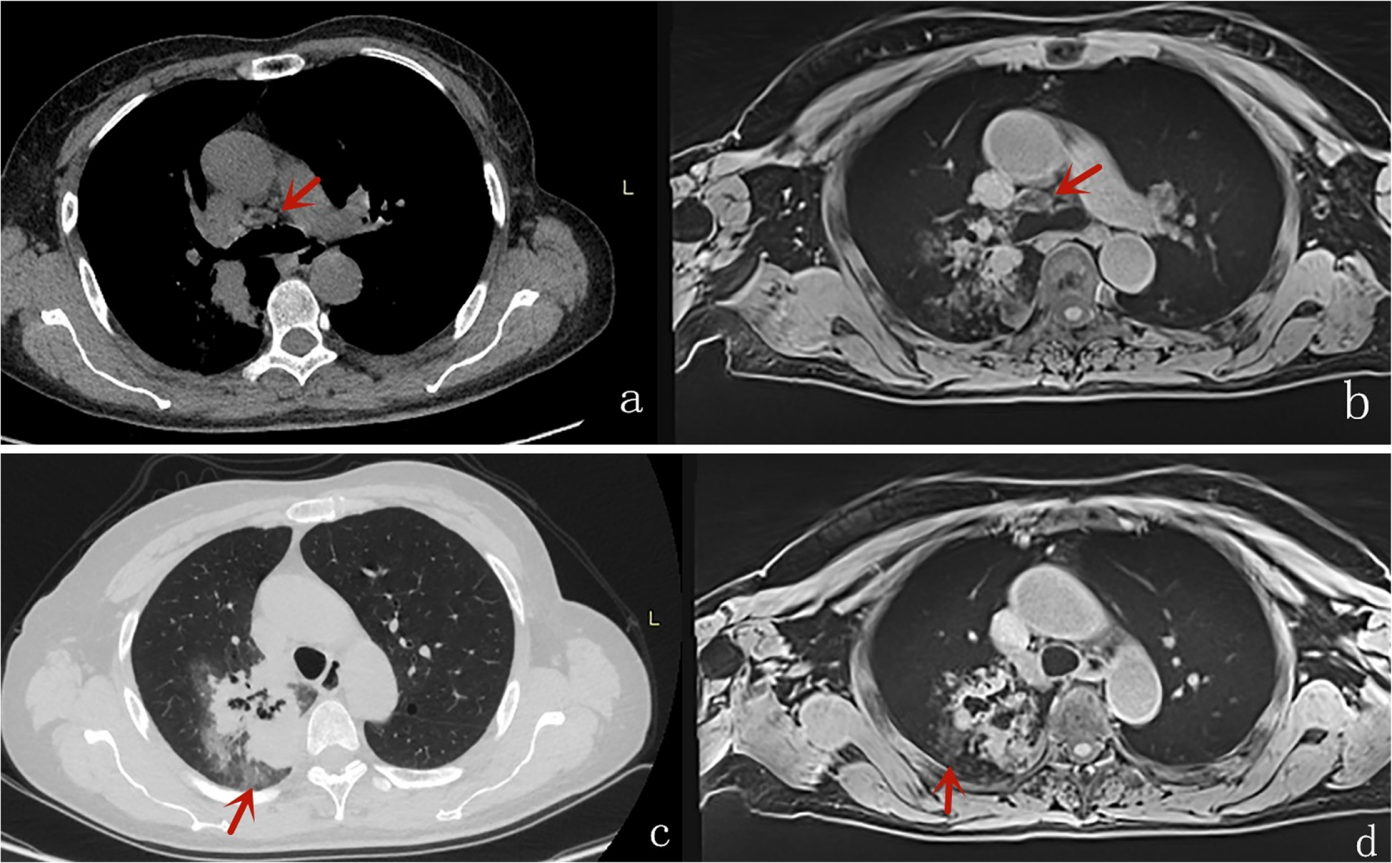
A female with acinar adenocarcinoma; axial CT (**A**) and r-VIBE (**B**) showed enlarged mediastinal lymph nodes (the red arrow). And sign of Halo was also visible in axial CT (**C**) and MR (**D**)

**Fig. 2:**
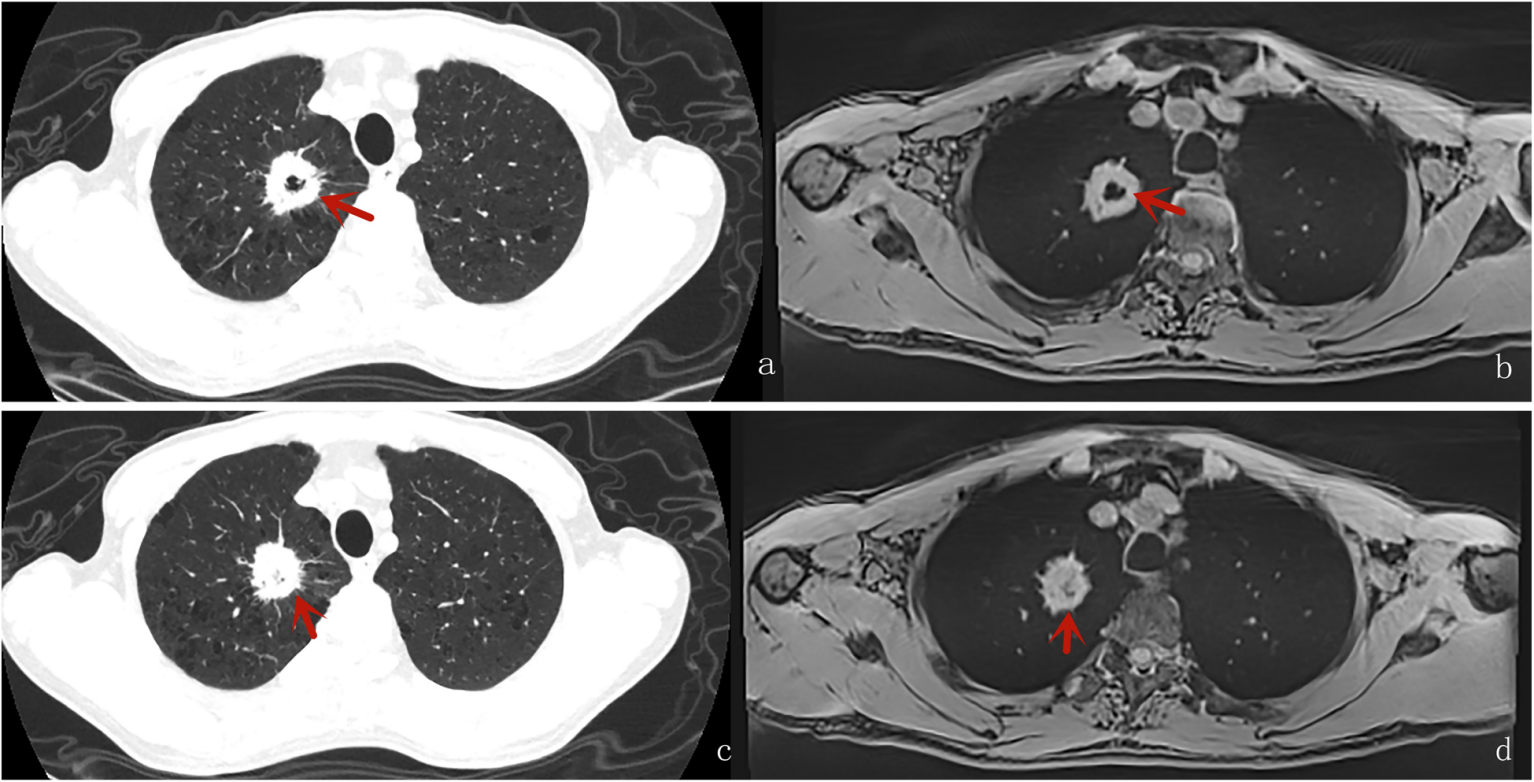
A male with adenocarcinoma; axial CT (**A**) and MR (**C**) showed cavitation (the red arrow); and sign of Spiculation was visible (the red arrow) in axial CT(**C**) and MR (**D**)

**Fig. 3:**
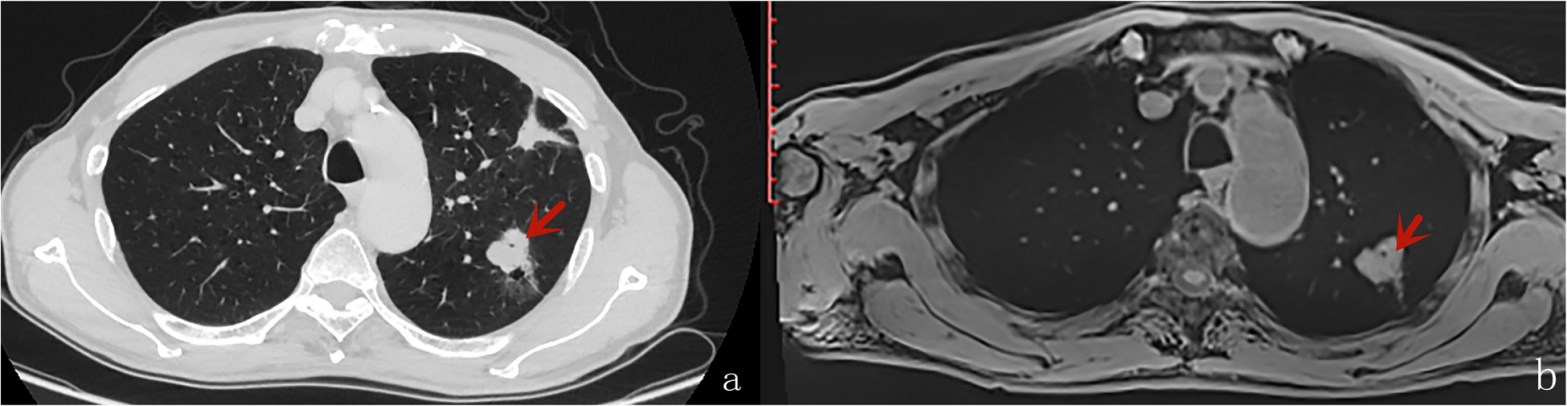
A male with small cell carcinoma, bubble-like attenuation was visible inside the lesion (the red arrow)in axial CT (**A**) and MR (**B**)

**Fig. 4:**
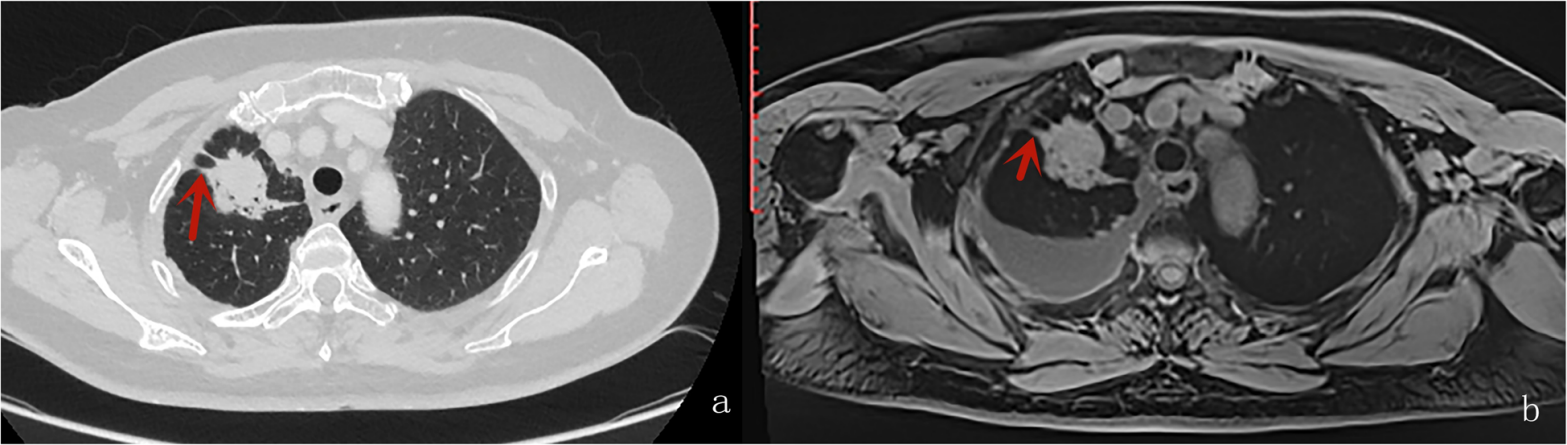
A female with adenocarcinoma; pleural indentation was found (the red arrow) in axial CT (**A**) and MR (**B**), the right pleural effusion was seen in r-VIBE obtained 1 day after CT

**Fig. 5:**
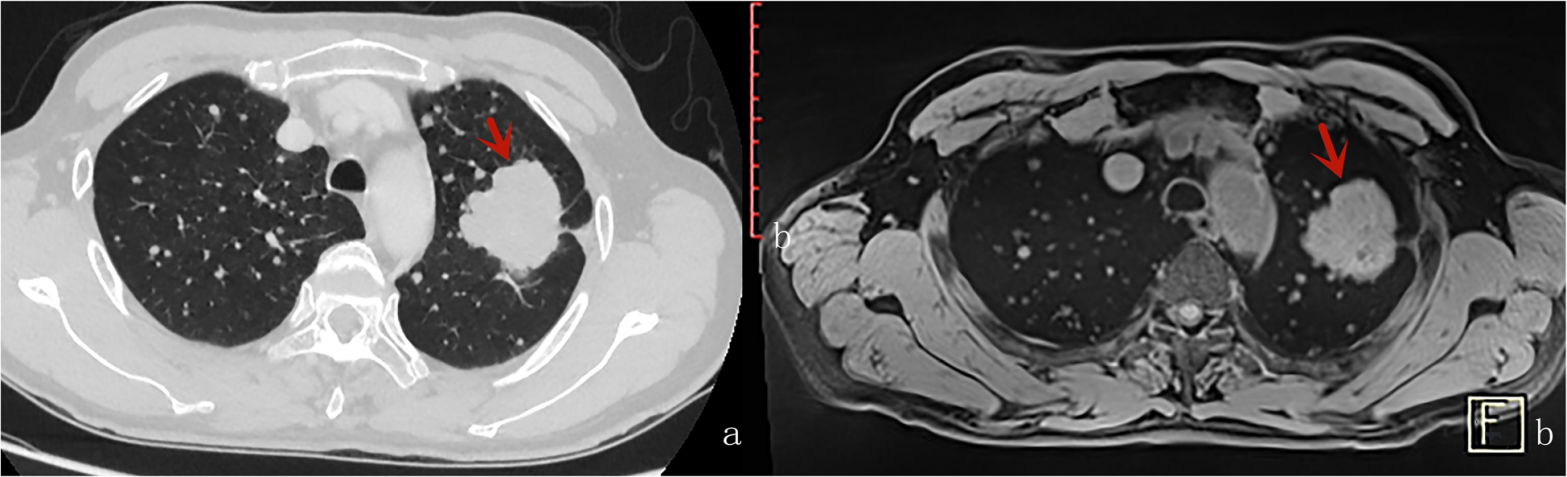
A male with adenocarcinoma, lobulation (the red arrow) and pleural indentation (the red arrowhead) were visible in axial CT(**A**) and MR (**B**)

Table 4 displays the results of inter-method agreement for the assessment findings of morphological characteristics by two methods. Inter-method agreements for the two methods (pulmonary MR imaging with r-VIBE and standard-dose CT) were significant and considered as “substantial” or “excellent” for evaluating following parameters: lobulation (*K* = 0.698; *P* < 0.0001), Spiculation (*K* = 0.605; *P* < 0.0001), Bubble-like attenuation (*K* = 0.700; *P* < 0.0001), convergence of vessels (*K* = 0.700; *P* < 0.0001). cavitation (*K* = 1.000; *P* < 0.0001), and mediastinal lymph node enlargement (*K* = 0.773; *P* < 0.0001); however, K values for inter-method agreements, which compared r-VIBE with standard-dose CT, were significant but “moderate”or “poor” for evaluating following parameters: Halo (*K* = 0.335; *P* = 0.002), pleural tag (K = 0.451; *P* < 0.0001), and calcification (K = 0.368; *P* < 0.0001) (Tables 4, Figs. 1, 2, 3, 4 and 5).

**Table 4:** Inter-method Agreement for Assessment of pulmonary lesion Findings Using two Methods

		Visual score						
**morphological characteristics**	**Methods**	**1**	**2**	**3**	**4**	**5**	**Kappa value**	***P*value**
**Lobulation**	Standard-dose CT vs.	25	0	0	0	22	0.698	<0.0001
	MR imaging with r-VIBE	27	0	1	2	17		
**Spiculation**	Standard-dose CT vs.	17	0	0	0	30	0.605	<0.0001
	MR imaging with r-VIBE	17	1	5	12	12		
**Pleural indentation**	Standard-dose CT vs.	23	0	0	0	24	0.451	<0.0001
	MR imaging with r-VIBE	28	0	3	10	6		
**Bubble-like attenuation**	Standard-dose CT vs.	36	0	0	0	11	0.700	<0.0001
	MR imaging with r-VIBE	37	1	3	1	5		
**Convergence of vessels**	Standard-dose CT vs.	26	0	0	0	21	0.700	<0.0001
	MR imaging with r-VIBE	32	0	0	3	12		
**Halo**	Standard-dose CT vs.	43	0	0	0	4	0.355	=0.002
	MR imaging with r-VIBE	45	1	0	0	1		
**Cavitation**	Standard-dose CT vs.	42	0	0	0	5	1.0	<0.0001
	MR imaging with r-VIBE	42	0	0	0	5		
**Calcification**	Standard-dose CT vs.	44	0	0	0	3	0.368	=0.010
	MR imaging with r-VIBE	45	0	0	0	2		
**Mediastinal lymph node enlargement**	Standard-dose CT vs.	30	0	0	0	17	0.773	<0.0001
	MR imaging with r-VIBE	28	0	1	1	17

## Discussion

In this study, we established that r-VIBE (MRI) sequence is a free-breathing method that can detect pulmonary nodules with satisfactory accuracy; therefore, r-VIBE (MRI) sequence is as an alternative imaging technique, which shows 100% sensitivity in detecting solid nodules with a diameter greater than 6 mm. Moreover, this technique shows92.3% sensitivity in detecting nodules, which have a diameter ranging from 4 to 6 mm. It is comparable to MCST in terms of its capability of maintaining image quality and visualization of morphological characteristics of lung lesions. Interobserver agreements for image quality and morphological diagnosis of lesions were significant and either substantial or excellent in this study. These results indicate that the findings of this study are reproducible. We analyzed each lesion with a maximum diameter of more than 10mm. The analyses suggested that pulmonary MR imaging with r-VIBE displayed radiological findings that had “substantial” or “excellent” agreement with most CT findings, except for halo, pleural indentation, and calcification. These fact indicated that r-VIBE has play as complementary role for evaluating morphological features of nodule or mass as compared with CT, especially for children, pregnant women and patients who need repeated reexamination of pulmonary nodules.

In a previous study, the MRI sequence for pulmonary nodule detection was perfectly concordant with the results obtained by CT. Compared with other sequences (T2 TSE, T2 SPIR, T2 STIR, T2 HASTE, and T1 out-of-phase), C-VIBE sequence shows highest detection rate (69%) and an ultra-short TE (< 1 ms) [12]. However, lungs can move upwards during free breathing. In particular, C-VIBE sequence can be used for detection only if lung cancer patients are able to hold their breath. Respiratory motion artifacts cause significant blurring of small lesions, limiting diagnostic accuracy.

r-VIBE is a 3D T1-weighted imaging sequence that performs rectilinear sampling in z direction and radial sampling in xy plane. In previous studies, pixel graininess was significantly lower in images obtained by r-VIBE. This is because r-VIBE has higher matrix and thinner slice thickness than C-VIBE. Using higher matrix, r-VIBE maximized the definition of lesion structures. Despite prolonged acquisition time, we easily added higher matrix as r-VIBE was a free-breathing sequence. In abdominal MRI scan, r-VIBE sequence produces images whose quality is similar to that of C-VIBE sequence. Chandarana et al. [21] reported that r-VIBE was better than C-VIBE in terms of overall image quality, including hepatic edge sharpness, hepatic vessel clarity, and respiratory motion robustness. However, another study of liver MRI reported that compared with C-VIBE, r-VIBE had lower but acceptable scores [22]. Our previous study also found that the r-VIBE correctly detected 94% of the pulmonary nodules as compared with CT. The detection rate increased to 100% for lesions≥6 mm. The C-VIBE had a lower overall detection rate (64.3%) of pulmonary nodules [23].

In the present study, we also found that compared to CT images, r-VIBE had significantly lower capability of diagnosing halo, pleural indentation, and calcification in each lesion. This suggests that r-VIBE (MRI) had limited capability of identifying calcification and those lesion with high proportion of gas. Moreover, poor inter-method agreement of “pleural indentation” may due to the difference of respiratory state between CT and MRI. The MR imaging with r-VIBE was applied on free-breathing condition. In contrast, thin-section CT scans were obtained when patients were capable of end-inspiration breath-holding. In addition, there were only four cases had the signs of halo, therefore, the limited sample number may affect the result of inter-method agreement in evaluating the sign of “halo”.

This study has some limitations. Firstly, pulmonary nodules detected by CT were only examined by MRI sequence; therefore, there were no ‘true negative’ findings in our study. Secondly, only visual analysis was used to evaluate the parameters of these nodules, including size and location. A 3D volume analysis of pulmonary nodules should be performed on an automated scale. Thirdly, there were limited cases had the signs of halo, cavitaion, therefore, the small sample number may affect the result of inter-method agreement in assessing these signs. Moreover, only one ground glass nodules were detected in this study, therefore, ground glass nodules detection rate were not considered in present study.

## Conclusions

In conclusion, pulmonary MR imaging with r-VIBE was useful in detecting lung nodules or masses as this technique produced high quality images.It is comparable to CT in the assessment of many morphological features despite some limitations.

## Data Availability

The datasets during and/or analyzed during the current study available from the corresponding author on reasonable request.

## Abbreviations

r-VIBE: radial 3D fat-suppressed T1-weighted gradient echo
CT: computed tomography
MRI: Magnetic Resonance Imaging
T2W-HASTE: T2 weighted half-fourier single-shot turbo spin echo
T1W-GRE: T1 weighted 3D gradient recall echo
UTE: Ultrashort echo time
C-VIBE: Conventional T1-weighted 3D gradient-echo volumetric interpolated breath-hold examination
ROC: Receiver operating characteristic
AUC: Area under the curve
